# Executive Functions Are Employed to Process Episodic and Relational Memories in Children With Autism Spectrum Disorders

**DOI:** 10.1037/a0034492

**Published:** 2013-11

**Authors:** Lara Maister, Jon S. Simons, Kate Plaisted-Grant

**Affiliations:** 1 Cambridge Laboratory for Research into Autism, Department of Experimental Psychology, University of Cambridge, Cambridge, United Kingdom; 2 Memory Laboratory, Department of Experimental Psychology, University of Cambridge, Cambridge, United Kingdom

**Keywords:** autism spectrum disorder, relational memory, hippocampus, posterior parietal cortex, executive functions

## Abstract

***Objective:*** Long-term memory functioning in autism spectrum disorders (ASDs) is marked by a characteristic pattern of impairments and strengths. Individuals with ASD show impairment in memory tasks that require the processing of relational and contextual information, but spared performance on tasks requiring more item-based, acontextual processing. Two experiments investigated the cognitive mechanisms underlying this memory profile. ***Method:*** A sample of 14 children with a diagnosis of high-functioning ASD (age: *M* = 12.2 years), and a matched control group of 14 typically developing (TD) children (age: *M* = 12.1 years), participated in a range of behavioral memory tasks in which we measured both relational and item-based memory abilities. They also completed a battery of executive function measures. ***Results:*** The ASD group showed specific deficits in relational memory, but spared or superior performance in item-based memory, across all tasks. Importantly, for ASD children, executive ability was significantly correlated with relational memory but not with item-based memory. No such relationship was present in the control group. This suggests that children with ASD atypically employed effortful, executive strategies to retrieve relational (but not item-specific) information, whereas TD children appeared to use more automatic processes. ***Conclusions:*** The relational memory impairment in ASD may result from a specific impairment in automatic associative retrieval processes with an increased reliance on effortful and strategic retrieval processes. Our findings allow specific neural predictions to be made regarding the interactive functioning of the hippocampus, prefrontal cortex, and posterior parietal cortex in ASD as a neural network supporting relational memory processing.

Autism spectrum disorders (ASDs) are a group of pervasive developmental disorders characterized by impairments in social interaction and communication, and the presence of stereotyped behaviors and restricted interests (American Psychiatric Association, 2000). In addition to these three core behavioral features of ASD, evidence has emerged to suggest that the memory abilities of individuals with ASD are also very different than those of typically developing (TD) individuals. Since Kanner’s (1943) earliest description of autism, it has often been reported that individuals with ASD have excellent rote memory; a startling ability to recite an entire bus timetable or a complete script from a TV program, for example, have become incorporated into the public’s popular perception of individuals with ASD. However, early empirical investigation revealed a complex pattern of both intact and impaired mnemonic abilities (Boucher & Warrington, 1976; Hermelin & O’Connor, 1967). More recent research involving individuals with ASD without learning difficulties suggests that there is a specific difficulty in processing relations between items in memory, but that they are unimpaired at processing more item-specific information (Bowler, Matthews, & Gardiner, 1997; Gaigg, Gardiner, & Bowler, 2008; Renner, Klinger, & Klinger, 2000; Smith, Gardiner, & Bowler, 2007; and see Boucher, Mayes & Bigham, 2012, for some differences in memory profile between high- and low-functioning individuals). A deficit in *relational* memory, as proposed by Bowler, Gaigg, and colleagues (e.g., Gaigg et al., 2008), can explain many of the features of memory performance in ASD.

For example, a relational memory deficit provides a ready explanation of the characteristic pattern of performance in ASD observed in laboratory-based free-recall tasks. Individuals with ASD perform at typical levels on *unrelated* free-recall tasks, in which the words to be remembered are semantically unrelated, but recall fewer items compared with TD individuals on *related* free-recall tasks, when the words can be semantically categorized (Boucher & Warrington, 1976; Bowler et al., 1997; Hermelin & O’Connor, 1967; Minshew & Goldstein, 1993; Renner et al., 2000; Tager-Flusberg, 1991; but see López & Leekam, 2003). TD individuals utilize the relational structure of the related list to aid their recall, and therefore perform better on related free-recall tasks than they do on unrelated free-recall tasks. Individuals with ASD do not appear to benefit from the relational structure of the list, and thus their recall on related free-recall tasks is lower than that of TD individuals.

Individuals with ASD also structure their responses in a different way than TD individuals. For example, when performing a related free-recall task, TD individuals tend to employ organizational strategies such as semantic clustering, whereby clusters of semantically related items are retrieved together (Bousfield, 1953). Individuals with ASD show markedly reduced semantic clustering and other subjective organization of items in recall tasks (Bowler, Gaigg, & Gardiner, 2008a; Gaigg et al., 2008; Minshew & Goldstein, 1993; Tager-Flusberg, 1991). A reduction or absence of recall of clusters of related items in ASD further demonstrates a reduced processing of relations between items in memory.

A relational memory deficit may also explain the reduced retrieval of contextual information often found in individuals with ASD (Bowler, Gaigg, & Gardiner, 2008b). This is clearly illustrated when analyzing performance of ASD individuals on tasks measuring autobiographical memory (ABM). Evidence suggests that the ABMs of individuals with ASD lack contextually specific episodic details, and contain more general and factual information than those of TD individuals (e.g., Crane & Goddard, 2008). This reduced recall of contextual detail, characteristic of ABM in ASD, could easily be explained by a relational memory deficit. To retrieve a vivid, contextually detailed ABM, the representation of an event must be retrieved as part of a coherent cluster of related contextual details. If these relations between items were not processed, ABMs would lack context and would consist of general, isolated event representations, characteristic of the overgeneral ABMs reported in ASD.

We argue that one possible mechanism underlying reduced utilization of relations between items in memory retrieval in ASD is suggested by Moscovitch’s (1992) distinction between associative and strategic retrieval. Moscovitch proposed that retrieval is mediated by two principal components: an automatic, associative component and a strategic, effortful component (Moscovitch, 1992; Moscovitch & Winocur, 2002). The associative component in Moscovitch’s model is involved in the automatic retrieval of associatively related representations, which he predicted was mediated by the hippocampus. In contrast, the effortful component of the model is involved in the conscious specification of cues, effortful memory search, and controlled monitoring processes postretrieval, and appears to be frontally mediated. Given that the automatic component of the model has an important role in retrieving associatively related items, it is likely to be critical for relational memory.

Support for this proposal comes from studies that have separated the roles of associative and effortful retrieval in relational memory performance. The majority of memory tasks employ both associative and effortful retrieval to varying extents; however, evidence suggests that the more the task relies on relational memory, the more performance can rely on automatic and associative retrieval processes. For example, a study by Cinan (2003) used a dual-task methodology to separate the effects of associative retrieval from effortful retrieval during related free-recall performance. A secondary task had a greater detrimental effect on retrieval of items from lists containing a larger number of semantic categories, suggesting that the initial cue specification of a semantic category name was more effortful than retrieving items within that category. This finding supports the dual-process account of related free-recall performance. First, a category concept is effortfully generated to act as a cue for items falling within that category. Second, this effortfully generated cue retrieves a number of semantically related items in an automatic, associative manner, demonstrating the critical role that automatic retrieval processes play in relational memory (Cinan, 2003).

This suggests that, in typical individuals, performance on relational memory tasks is more heavily reliant on the associative retrieval component compared with performance on item-based memory tasks. We argue that the relational memory deficit in ASD may result from a reduction in automatic associative retrieval of related items in memory, thought to be mediated by the hippocampus. If this is the case, it is possible that individuals with ASD may have to rely more on effortful, controlled retrieval processes during relational memory tasks to compensate for a deficit in associative retrieval. If individuals with ASD retrieved a greater proportion of items individually and effortfully, due to a reduction of automatic, associative retrieval of related items, this could explain both their impaired performance on related free-recall tasks, and their spared performance on unrelated free-recall tasks. It could also explain the absence of typical semantic clustering observed in related free-recall performance in ASD.

Further, a specific impairment in hippocampally mediated, associative retrieval processes with an increased reliance on effortful, frontally mediated retrieval processes might also account for the pattern of impairment and strength seen in autobiographical recall in ASD. In a similar manner to related free-recall performance, the recall of ABMs is thought to involve two sequentially employed retrieval processes (Conway & Pleydell-Pearce, 2000). Autobiographical recall involves effortful cue generation (usually, general event details and factual information), which then can go on to automatically activate a cluster of episodic, associatively related contextual details. A study by Piolino et al. (2008) identified frontal areas (specifically, the left medial orbital frontal regions) as mediating the effortful retrieval of general autobiographical details, and the right hippocampus as mediating the retrieval of specific episodic context (see also Addis, Moscovitch, Crawley, & McAndrews, 2004). This not only provides neural evidence for the two distinct processes postulated by Conway and Pleydell-Pearce (2000) but also ties the two processes to the frontal regions and hippocampus, as was predicted by Moscovitch and Winocur (2002). Again, this suggests that relational memory—in this case, essential for the retrieval of episodic, associatively related contextual details—is reliant on automatic, associative retrieval processes mediated by the hippocampus. If individuals with ASD have reduced automatic, associative retrieval of related items in memory, and correspondingly rely more heavily on effortful, controlled retrieval processes, it could explain the characteristic pattern of a reduction in the recall of specific episodic context, and an increase in the recall of general and factual details seen in the ABM of individuals with ASD.

In summary, we argue that in situations in which the utilization of associative relations between items is important for retrieval, individuals with ASD are less able to recruit automatic, associative retrieval processes in the same way as TD individuals. This may be a direct result of impairment in the associative, automatic retrieval of items from episodic memory, and could be related to hippocampal abnormalities. During relational memory tasks, this reduced level of associative retrieval in ASD may be accompanied by a corresponding overreliance on controlled, frontally mediated retrieval processes. If this were the case, retrieval in these situations would be slower, more effortful, and less successful than that of TD individuals. Importantly, this raises the possibility that the success of such retrieval in ASD is reliant on *individual* executive ability; those with poor cognitive control may be more impaired in relational memory tasks than those who have better cognitive control.

The current study aimed to test this suggestion by assessing relational and item-based memory in children with ASD in two experiments. In the first, mnemonic processing was assessed in both a seminaturalistic ABM task and more tightly controlled standard laboratory tasks of related and unrelated free recall. In the second experiment, we examined our prediction further by assessing both relational and item-based retrieval, but within a single task. The adoption of a single task for this purpose removed any possibility of the adoption of specific strategies between separate tasks of item-based and relational memory retrieval, which might have influenced our results in the separate related and unrelated free-recall tasks of our first experiment. A small battery of executive function (EF) measures was employed in each experiment to assess effortful cognitive control abilities. As we had no a priori hypothesis about the role of any specific EF, we selected a representative range of EF tests that are well-established measures in the literature. A correlational analysis assessed the role of these controlled, executive abilities in the performance of relational versus item-based recall.

## Experiment 1

TD children and children with ASD were given an ABM task and two free-recall tasks, one using related, and the other using unrelated, information. In the ABM task, we predicted that children with ASD would display the previously observed pattern of relatively good retrieval of general, nonepisodic autobiographical information compared with specific episodic information. Further, we predicted that specifically episodic, but not general, autobiographical information would be retrieved in ASD by atypically employing effortful, controlled retrieval processes, whereas TD individuals would rely more on automatic, associative retrieval. Similarly, we expected to observe the pattern of reduced recall of items in the related free-recall test, but unimpaired recall in the unrelated free-recall test, and predicted an atypical employment of effortful retrieval processes only in the related free-recall task in the ASD group. We therefore predicted selective relationships between performance on the EF tasks and recall of episodic, but not general, details in the ABM task, and performance in the related, but not the unrelated, free-recall task.

### Method

#### Participants

Fourteen children with ASD (the ASD group) and 14 TD children (the TD group) participated in the study. The children were between 11 and 13 years old. Diagnoses for the ASD group (14 boys) were made by an experienced, trained independent clinician and were based on the Autism Diagnostic Interview–Revised (ADI-R; Lord, Rutter, & Couteur, 1994), which focuses on the following three areas: communication and language, reciprocal social interaction, and repetitive and restricted behaviors. Children with additional psychiatric diagnoses were excluded, as established by referring to Statements of Special Educational Needs. None of the children were taking medication. The children in the TD group (13 boys, 1 girl) had no known psychiatric diagnoses, and were compared with the ASD group on measures of chronological age, verbal ability, and nonverbal IQ. Verbal ability was assessed using the British Picture Vocabulary Scale (BPVS; Dunn, Whetton & Pintilie, 1988) and nonverbal IQ was measured using Raven’s Standard Progressive Matrices (RPM; Raven, Court & Raven, 1977). Two-tailed independent *t* tests confirmed that the children in the TD group did not significantly differ from the children in the ASD group on chronological age, BPVS score, or RPM score. Results of these analyses are summarized in Table 1. Despite the ASD and TD groups not differing significantly on any of these measures, the ASD group had marginally lower BPVS scores than the TD group (*p* = .10), and so variation in BPVS score was statistically controlled for in subsequent correlational analyses. Analysis of BPVS, RPM, and age data were carried out prior to the analysis of test data, and no child was excluded at this point or substituted for another child.[Table-anchor tbl1]

All children were recruited from mainstream primary and secondary schools in Surrey, Derbyshire, and Shropshire, United Kingdom. Informed parental consent was obtained for each child and the study was approved by the University of Cambridge Psychology Research Ethics Committee.

#### Measures

Two memory tasks were carried out, each of which provided a measure of relational memory and a measure of item-based memory. The tasks consisted of an ABM task and a free-recall task. Additionally, a small battery of four EF measures was taken to assess core executive processes, including set shifting, generativity, maintenance, and inhibition. Finally, psychometric measures were employed to provide information about the participants’ general levels of functioning.

##### ABM task

The children were asked to verbally recall three ABMs. They were instructed to choose three distinct personal past events that they could clearly remember, and report as much about what happened as possible. The instructions were designed to elicit narratives of three specific autobiographical events salient to the child. Three example memory cues were given: a birthday party, a school trip, and something they did on a holiday. The children could choose a personal memory relating to one of these three general cues, or produce their own choices of events, with no time limit.

The responses were recorded and scored in accordance with an adapted version of the Autobiographical Interview (Levine, Svoboda, Hay, Winocur, & Moscovitch, 2002). The responses were segmented into separate details, defined by Levine et al. as “a unique occurrence, observation, or thought, typically expressed as a grammatical clause (i.e., a subject and predicate)” (p. 679). Each detail was categorized as one of two types: a general detail (G.ABM) or an episodic detail (E.ABM). Recall of G.ABMs served as the item-based memory measure, and recall of E.ABMs served as the relational memory measure. G.ABMs were defined as those that did not have a specific spatiotemporal context. These included details that formed part of the general narrative structure, but in themselves did not contain specific contextual information (for example, “We went on holiday to France”), and also included factual information that was not spatiotemporally tied to the described event (for example, “My sister loves croissants”). E.ABMs were defined as specific perceptual, emotional, or thought utterances that were specific to the spatiotemporal context of the described event, for example “She had a black and brown dog in the kitchen,” “I was scared,” and “I thought it would bite me.” Utterances that did not fall in either of these categories, such as metacognitive statements (e.g., “I can’t really remember …”), repetitions of details, or other utterances unrelated to the task were not scored. Only details forming the general narrative (general event details) were explicitly requested in the verbal instructions the children received; therefore, it was assumed any E.ABM reported during the narratives were incidentally retrieved and not effortfully obtained.

##### Free-recall tasks

The free-recall task contained two conditions; in one condition, the children had to learn and recall a list of semantically unrelated words (the unrelated condition), and in the other condition, they had to learn and recall a list of words that could be semantically categorized (the related condition). Performance in the unrelated condition served as the item-based memory measure, and performance in the related condition served as the relational memory measure. Both conditions were conducted on a 15-in. LCD Acer laptop running Microsoft Windows XP, and were programmed using DMDX software (Forster & Forster, 2003).

In both conditions, children were informed that they would be presented with a list of words that they must try to remember, as they were subsequently going to be asked to recall as many as possible. For the unrelated condition, 15 semantically unrelated words were presented to the participant on a computer screen. Each word was presented individually for 3 s, with a 1-s gap between presentations. After the last word had been displayed, the children were engaged in conversation about their favorite school subject for 1 min. Participants were then asked to recall as many of the words as possible, out loud and in any order. Responses were recorded on a voice recorder for later analysis. The procedure in the related condition was identical to that of the unrelated condition, but the words to be remembered were selected from three semantic categories; fruit, transport, and clothing. Five words from each category were used. Each word had a maximum age of acquisition of 5 years old (Stadthagen-Gonzalez & Davis, 2006) and were selected from Van Overschelde, Rawson, and Dunlosky’s (2004) updated version of the Battig and Montague norms. Association coefficients were calculated using latent semantic analysis (LSA, Landauer, Foltz, & Laham, 1998). LSA is a computational model of semantic space, based on the statistical analysis of a large text corpus. Each category had an average association coefficient of .32 or higher. The words were presented in a pseudorandom order, ensuring that no two words from the same category appeared consecutively. After the last word had been displayed, the children were engaged in conversation about their favorite school subject for 1 min. As in the unrelated condition, their recall responses were recorded on a voice recorder for later analysis.

##### EF tasks

Four measures of EF were carried out to give a general overview of the participants’ executive abilities. The Spatial Span (SSP) task from the Cambridge Neuropsychological Test Automated Battery (CANTAB) was used to assess working memory capacity. It was carried out on a 15-in. touch-screen laptop. The task consisted of a set of white squares shown on the screen. Some of these squares briefly changed color in a variable sequence. The participant was required to touch the boxes that changed color in the same order that they were displayed by the computer. The number of boxes in the sequence increased from two at the start of the test to nine at the end. The maximum number of boxes correctly responded to was recorded.

The Intra–Extra Dimensional (ID/ED) set shift task was used to assess flexibility and set shifting. This is a computerized analogue of the Wisconsin Card Sort Test from the CANTAB battery. The children learned a series of 9 two-alternative, forced-choice discriminations using feedback provided automatically by the computer. Touching the correct stimulus resulted in the word “correct” displayed on the screen, before the next trial began. If the participant touched the wrong stimulus, the computer displayed the word “incorrect.” Throughout the task, they were required to learn rules, initially through trial and error. Once the rule is achieved on six consecutive occasions, the computer establishes a new rule. At each stage of the test, two sets of visual stimuli are displayed. Two critical shifts occur during the test, one at the sixth rule change, when subjects must shift to new exemplars, and a second at the eighth rule change, in which subjects must make an extradimensional shift to attending to a second dimension that had previously been irrelevant. The total number of errors was recorded for each child, adjusted for number of trials completed. Additionally, the number of errors in the set requiring an extradimensional shift was also recorded.

A phonemic verbal fluency task (Benton, 1968) and a semantic verbal fluency task (Western Aphasia Battery; Kertesz, 1982) were used to provide an overall measure of verbal fluency. For the phonemic fluency task, children were required to produce as many words as possible beginning with the letter “B.” For the semantic fluency task, they were asked to name as many items as possible from the category “animals,” Children had 90 s in which to respond for each category, and responses were recorded on a voice recorder for later analysis. Performance in both verbal fluency tasks were combined to provide a composite measure of general verbal fluency.

To assess inhibition, a Stroop task was employed. We used a shortened, computerized version of the classic Stroop Task (Stroop, 1935) from the Psychology Experiment Building Language battery (Mueller, 2010). Participants used the keyboard to respond. Each of four keys corresponded to each of four colors; red, green, blue, and yellow. This correspondence was displayed at the bottom of the screen throughout the task. The task consisted of two response blocks. In the first—name reading—the participant was required to read the word in the center of the screen (either red, blue, green, or yellow) and press the corresponding key on the keyboard. For the second response block—color identification—the participant was asked to ignore what the word said but instead press the key corresponding to the color the word was printed in. Trials were either consistent (when the name of the word and the color of the letters matched) or inconsistent (when the name of the word and the color it was printed in did not match). An interference score was obtained by calculating how much faster the color-identification responses were than the name-reading responses in inconsistent trials, taking into account performance speed on consistent trials.

##### Psychometric measures

The RPM and BPVS were administered according to the standardized testing procedures. The BPVS was scored using published norms (Dunn, Whetton, & Pintilie, 1988) to obtain standardized scores independent of age.

#### General procedure

Children completed two separate testing sessions, the first lasting 1 hr and the second lasting roughly 45 min. The sessions were conducted on separate days and took place individually in quiet rooms at their school. The BPVS, RPM, Stroop, and verbal fluency tasks were completed during the first session. The SSP task, the ID/ED set shift task, the free-recall tasks, and the ABM task were carried out in the second session. The order of the sessions was counterbalanced across participants, and the order of tasks within the sessions was randomized.

### Results

For all analyses, the alpha level was set at .05. The statistical tests were carried out using IBM SPSS 18.0 statistical software.

#### ABM

Responses were scored following the procedure of Levine et al. (2002) by the first author, who was blind to group membership. An additional two independent raters, who were blind to both group membership and hypothesis, each scored a randomly selected 85% subset of the responses to assess interrater reliability. Intraclass correlation coefficients showed good reliability using Fleiss’s (1973) benchmarks: .40 to .75, fair to good, and >.75, excellent. Reliability for the G.ABM variable was good, .66, as was reliability for the episodic (E.ABM) details, .71.

**Figure 1 fig1:**
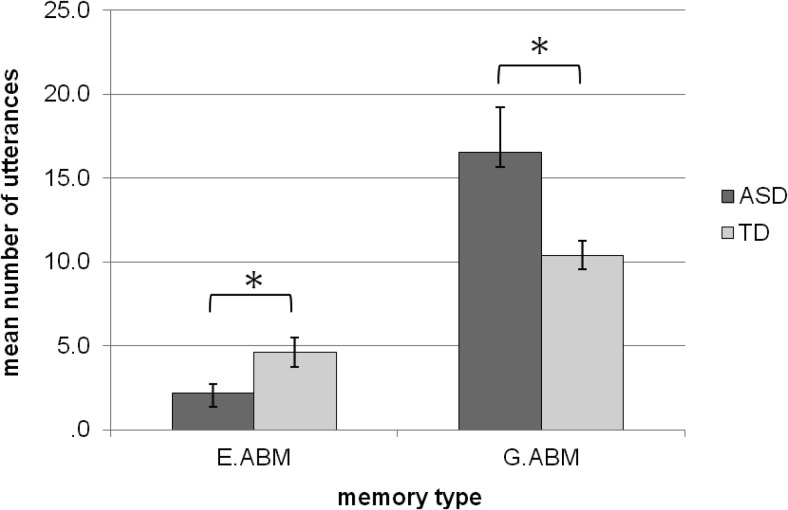
Chart showing mean utterances categorized as general details (G.ABMs) and episodic details (E.ABMs) for both the TD group and ASD group. Error bars reflect the standard error of the means. Asterisks denote significant group difference at *p* < .05.

The memory responses were analyzed using a 2 (group: ASD vs. TD) × 2 (memory type: G.ABM vs. E.ABM) repeated measures ANOVA. There was no significant main effect of group, *F*(1, 26) = 0.57, *p* = .46, but there was a main effect of memory type, *F*(1, 26) = 48.22, *p* < .001, with the participants uttering more G.ABMs (*M* = 12.62, *SD* = 7.21) than E.ABMs (*M* = 3.52, *SD* = 2.89). There was also a Memory Type × Group interaction, *F*(1, 26) = 6.88, *p* = .015. Simple effects analysis revealed that the ASD group uttered significantly fewer E.ABMs (*M* = 2.31, *SD* = 1.87) than the TD group (*M* = 4.64, *SD* = 3.27), *t*(26) = 2.25, *p* = .03. Conversely, the ASD group uttered a significantly greater number of G.ABMs (*M* = 16.54, *SD* = 10.12) than the TD group (*M* = 10.39, *SD* = 3.21), *t*(15.58) = −2.17, *p* = .046. These results are illustrated in Figure 1.[Fig-anchor fig1]

#### Free recall

**Figure 2 fig2:**
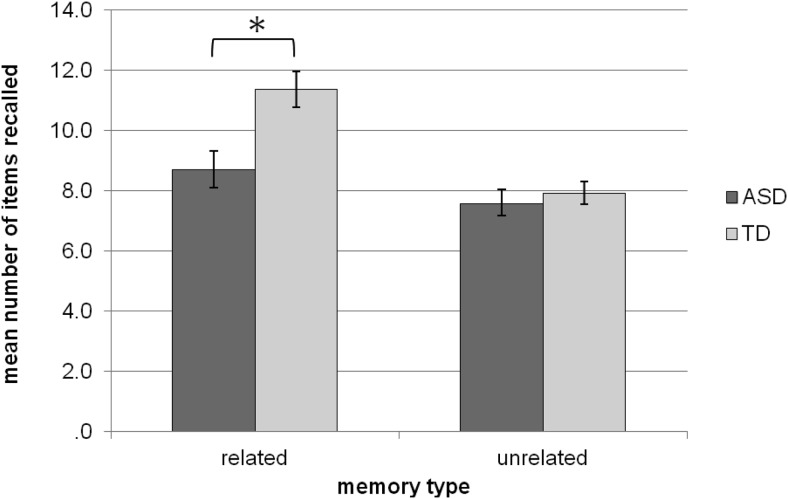
Chart showing mean number of recalled items in the related and unrelated free recall tasks, for the TD group and ASD group. Error bars reflect the standard error of the means. Asterisks denote significant group difference at *p* < .05.

The recall score reflected the number of words correctly recalled from the list of 15 items. A repeated measures 2 (memory type: related vs. unrelated) × 2 (group: ASD vs. TD) ANOVA was used to analyze the recall scores. There was a main effect of group, *F*(1, 26) = 7.81, *p* = .01, with the ASD group recalling significantly fewer words (*M* = 8.14, *SD* = 1.63) than the TD group (*M* = 9.64, *SD* = 1.16). There was also a main effect of memory type, *F*(1, 26) = 19.49, *p* < .001, with the related memory condition yielding overall higher recall scores (*M* = 10.04, *SD* = 2.62) than the unrelated condition (*M* = 7.75, *SD* = 1.58). This was modulated by a Group × Memory Type interaction, *F*(1, 26) = 4.87, *p* = .036, as illustrated in Figure 2. Independent-samples *t* tests revealed that the ASD group recalled significantly fewer words (*M* = 8.71, *SD* = 2.33) than the TD group (*M* = 11.36, *SD* = 2.24) in the related recall condition, *t*(26) = 3.06, *p* = .005. Conversely, there was no significant difference between ASD (*M* = 7.57, *SD* = 1.74) and TD (*M* = 7.93, *SD* = 1.44) performance in the unrelated recall condition, *t*(26) = 0.59, *p* = .559.[Fig-anchor fig2]

#### EF

Group performance on the SSP measure was assessed by analyzing span length (the longest sequence successfully recalled) using a univariate ANOVA with group (ASD vs. TD) as a factor. There was no significant difference in performance between groups in this task (ASD: *M* = 6.92, *SD* = 1.19; TD: *M* = 7.00, *SD* = 1.24), *F*(1, 26) = 0.03, *p* = .87.

In the ID/ED task, the total number of errors was analyzed using a univariate ANOVA with group (ASD vs. TD) as a factor. There was again no significant difference in performance between groups in this task (ASD: *M* = 20.23, *SD* = 14.28; TD: *M* = 17.86, *SD* = 10.54), *F*(1, 26) = 0.03, *p* = .52. An identical analysis was performed on the number of errors in the extradimensional shift only, and again no group difference was found (ASD: *M* = 8.42, *SD* = 9.23; TD: *M* = 6.00, *SD* = 7.46), *F*(1, 26) = 0.55, *p* = .47. Total number of errors correlated very strongly with number of errors on the extradimensional shift trial (*r* = .92, *p* < .0001), and so to simplify later correlational analysis, only total errors were used.

To assess verbal fluency, the number of correct responses given within the 30-s time period were analyzed using a univariate ANOVA with group (ASD vs. TD) as a factor, for both semantic fluency and phonemic fluency tasks. There were no significant group effects in the semantic fluency task, *F*(1, 26) = 0.94, *p* = .34, nor the phonemic fluency task, *F*(1, 26) = 0.96, *p* = .34. A composite fluency score was then calculated by summing both semantic and phonemic scores. The groups did not differ on this measure (ASD: *M* = 11.18, *SD* = 2.88: TD: *M* = 12.43, *SD* = 2.51), *F*(1, 26) = 1.50, *p* = .23.

To assess performance in the Stroop task, an interference score (calculated as described in the Method section) was analyzed using a univariate ANOVA with group (ASD vs. TD) as a factor. As in the other tests of EF, no significant difference between groups was found (ASD: *M* = 200.61, *SD* = 750.65; TD: *M* = 312.07, *SD* = 426.08), *F*(1, 26) = 0.23, *p* = .64.

#### Correlational analyses

To investigate the roles that EF may have played in the memory tasks in both ASD and TD groups, a correlational analysis was employed. Given that a high score in the ID/ED task reflects poor performance, whereas high scores in other measures reflect good performance, to facilitate interpretation, the direction of correlation coefficients involving the ID/ED task were inverted so that a positive coefficient reflected a positive relationship between levels of performance on any measure. First, the relationships between the psychometric measures (BPVS and RPM) and performance on the memory and EF measures were analyzed. Spearman’s correlation coefficients showed no significant relationships for any of the experimental tasks (*p*s > .05). We then analyzed the relationships between performance on the EF measures and performance on the memory measures. Because the ASD group had marginally lower BPVS scores than the TD group in this experiment (*p* = .10), any analyses yielding significant correlations were repeated using a partial correlational analysis using BPVS score as covariate. This ensured that any correlations between memory performance and EF performance were not due to a common relationship with verbal ability. No data points fell outside the *M* ± 2*SD* criterion for identification of outliers; thus, all participants were included in all analyses.

##### ABM

In the ASD group, recall of episodic ABM was significantly correlated with performance on the ID/ED task (*r* = .60, *p* = .03), and this correlation remained significant when BPVS was controlled for (*r* = .70, *p* = .01). The better the children with ASD were at the executive task, the more E.ABMs they recalled in the ABM task. In contrast, recall of general ABM details were not related to any executive measure (*p*s ≥ .41). In the TD group, there were no significant correlations between executive performance and either ABM measure (*p*s ≥ .49).

To further understand the role of ID/ED performance in episodic ABM recall, the children in the ASD group were divided into high- and low-ID/ED-performance subgroups, depending on whether their score fell below or above the median for the entire sample of ASD and TD children (*Mdn* = 14.0). The ASD children in the high-ID/ED subgroup (*n* = 7) were not significantly impaired in episodic ABM recall, reporting a comparable number of E.ABMs with the TD group (*n* = 14; ASD: *M* = 3.42, *SD* = 1.76; TD: *M* = 4.64, *SD* = 3.27), *t*(19) = 0.91, *p* = .374. In contrast, the ASD children in the low-ID/ED subgroup (*n* = 7) recalled significantly fewer E.ABMs (*M* = 1.00, *SD* = 0.89) compared with the TD children, *t*(19) = 3.84, *p* = .001. However, Neither ASD subgroup differed when compared with the TD group on age, RPM score, or BPVS score (*p*s > .05).

##### Free recall

In the ASD group, recall score in the related condition was positively correlated with performance on the SSP task (*r* = .70, *p* = .005), and this correlation remained significant when BPVS score was controlled for (*r* = .60, *p* = .038). The better the children with ASD performed on the SSP task, the more words they recalled in the related condition of the free-recall task. This relationship was not present for unrelated free-recall performance (*r* = .13, *p* = .658). In the TD group, there were no significant correlations between executive performance and either free-recall measure (*p*s ≥ .43).

Again, to further understand the role of SSP performance in related free recall, the children in the ASD group were divided into high- and low-SSP-performance subgroups, depending on whether their SSP score fell below or above the median for the entire sample (*Mdn* = 7.0). The ASD children in the high-SSP subgroup (*n* = 7) were not significantly impaired in related free recall, retrieving a similar number of words as the TD group (*n* = 14; ASD: *M* = 10.00, *SD* = 0.89; TD: *M* = 11.36, *SD* = 2.24), *t*(19) = 1.41, *p* = .136. In contrast, the ASD children in the low-SSP subgroup (*n* = 7) recalled significantly fewer words (*M* = 7.71, *SD* = 2.87) compared with the TD children, *t*(19) = 2.94, *p* = .015. Again, neither ASD subgroup differed when compared with the TD group on age, RPM score, or BPVS score (*p*s > .05).

## Experiment 2

In Experiment 1, we measured relational and item-based memory in a naturalistic task, and more tightly controlled lab-based analogue tasks (unrelated and related free recall). Although the same pattern of results was obtained for the seminaturalistic and laboratory tasks (poorer relational memory compared with item-based memory in the ASD group), there was a further critical methodological difference between them: In the first, seminaturalistic task (ABM recall), the two memory measures were obtained from the same task, whereas each measure was drawn from one of two tasks in the lab-based recall tasks. Thus, although the relative contribution of either type of memory to task success was not clear to the participant in the ABM task, it is certainly feasible that it may have been in the laboratory free-recall tasks. In order to obtain measures of the two types of memory within controlled laboratory procedures that prevent the adoption of a specific strategy, we used a category memory task, an adapted version of a free-recall task devised by Hunt and Seta (1984) and applied to memory research in ASD by Gaigg et al. (2008). The task comprised a learning phase of 16 words and an immediate recall phase. Of the 16 words, nine were drawn from one category (e.g., animals) and served as the large category, whereas two other sets (five words from the category “fruit” and two words from the category “clothing”) served as small categories. Words from each category were randomly interspersed with words of the other categories during the learning phase. The assumption of the task is that recall of items from a list of categorized words depends on the availability of both relational information and item-based information. In small categories containing few examplars, relational information is relatively unobvious, and thus recall of items from the small category will disproportionally benefit from the encoding of relational information. Conversely, in large categories containing many exemplars, item-based information differentiating the exemplars is relatively unobvious, and thus the encoding of item-based information will disproportionately benefit the retrieval of items from large categories (Hunt & Seta, 1984).

The proportions of items recalled from the two small categories (two and five words) were grouped to form a “relational” recall score, whereas the proportion of recalled items from the remaining large (nine word) category formed the item-based recall score. We predicted that children with ASD would obtain a lower relational recall score than comparison children, whereas no group differences would be observed on the item-based recall score, replicating Gaigg et al. (2008). Further, the two measures of individual EF ability that had shown correlations with memory measures in the ASD group in Experiment 1 (ID/ED and SSP) were used, and, as in Experiment 1, we predicted that, due to the effortful recruitment of strategic processes for recalling related information by the ASD children, selective correlations between the relational recall score, but not the item-based score, and EF ability would be seen only in the ASD group.

### Method

#### Participants

Fourteen children with ASD and 14 TD children participated. The children were between 9 and 14 years old and had not participated in the previous experiment. The children in the ASD group (13 boys, one girl) had all received formal diagnoses of an ASD from an experienced, trained independent clinician, based on the ADI-R (Lord, Rutter, & Couteur, 1994). Children with additional psychiatric diagnoses were excluded, as established by referring to Statements of Special Educational Needs. None of the children were taking medication. The children in the TD group (11 boys, three girls) had no known psychological diagnoses and were matched with the ASD group on chronological age, verbal ability, and nonverbal reasoning. Verbal ability was assessed using the BPVS, and nonverbal reasoning was measured using RPM.

All children were recruited from mainstream primary and middle schools in England and Wales. The children in the ASD group were all attending schools with a specialist autism provision. Informed parental consent was obtained for each child, and the Cambridge Psychology Research Ethics Committee approved the study. All psychometric data for the participants are summarized in Table 2.[Table-anchor tbl2]

#### Measures

In this experiment, a single task was used to assess both relational and item-based memory. EF ability was assessed using two tasks; selection was based on the results of Experiment 1 as those identified as playing a role in memory in ASD. Finally, psychometric measures were taken to provide information about general functioning.

##### Category memory task

During an initial learning phase, 16 words were presented to the participant on a computer screen. The words were selected from three semantic categories: animals, fruit, and clothing. Nine words from the category “animals” were selected from the Van Overschelde et al. (2004) norms and served as the large category. Five words from the category “fruit” and two words from the category “clothing” were selected to act as small categories. The items in each category had an average frequency of response of 0.50 for the animal category, 0.70 for the fruit category, and 0.88 for the clothing category (see Van Overschelde et al., 2004, for details). Word presentation was randomized to ensure that no words consistently occupied primacy or recency positions, and that presentation order was different for each participant. Each word was presented individually for 3 s on a 15-in. laptop computer screen in 48-point Arial font, with a 1-s interval between presentations. Participants were then asked to recall as many of the words as possible out loud, in any order. Responses were recorded for later analysis.

##### EF tasks

The EF tasks (the ID/ED and SSP tasks from the CANTAB battery) were carried out on a 15-in. touch-screen laptop. The procedures were identical to those used in Experiment 1.

##### Psychometric measures

RPM and BPVS were administered according to the standardized testing procedures. The BPVS was scored using published norms (Dunn et al., 1988) to obtain standardized scores independent of age.

#### General procedure

Children completed two separate testing sessions, the first lasting roughly 45 min and the second lasting 35 min. The sessions were conducted on separate days and took place individually in quiet rooms at their school. The BPVS and RPM tasks were completed during the first session. The SSP task, the ID/ED set shift task, and the category memory task were carried out in the second session. The order of the sessions was counterbalanced across participants, and the order of tasks within the sessions was randomized.

### Results

For all analyses, the alpha level was set at .05. The statistical tests were carried out using IBM SPSS 18.0 statistical software, and two-tailed tests were used throughout. No data points fell outside the *M* ± 2*SD* criterion for identification of outliers, and thus all participants were included in all analyses.

#### Category memory task

**Figure 3 fig3:**
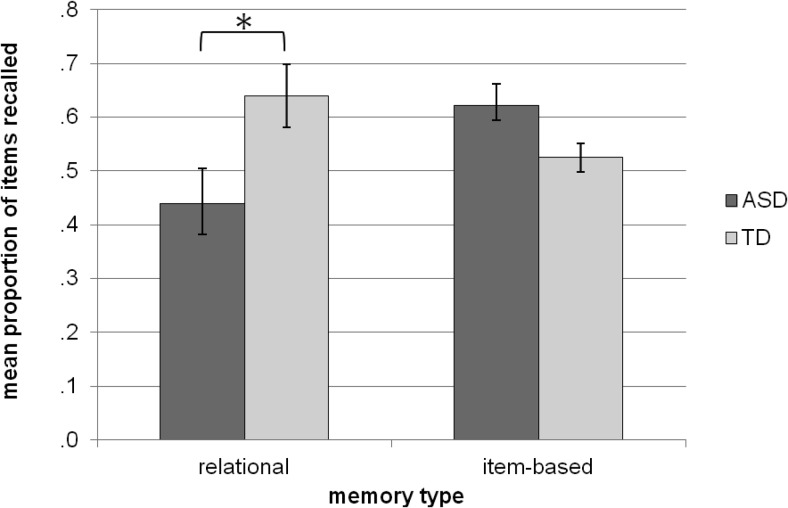
Chart showing mean proportion of words recalled from relational section of task and item-based section of task for both the TD group and ASD group. Error bars reflect the standard error of the means. Asterisks denote significant group difference at *p* < .05.

To analyze performance on the category memory task, the proportions of items recalled from the two small categories (two and five words) were grouped to form a relational recall score. The proportion of recalled items from the remaining large (nine word) category formed the item-based recall score. Recall scores were then analyzed using a 2 (group: ASD vs. TD) × 2 (memory type: relational vs. item-based) repeated measures mixed ANOVA. There was no main effect of memory type, *F*(1, 26) = .28, *p* = .60, and no main effect of group, *F*(1, 26) = 2.40, *p* = .13. However, there was a Group × Memory type interaction present, *F*(1, 26) = 5.66, *p* = .03. Simple effects analysis revealed that the ASD group had a lower relational recall score (recalling a smaller proportion of words from the two small categories) than did the TD group (ASD: *M* = .44, *SD* = .24; TD: *M* = .64, *SD* = .22), *F*(1, 26) = 5.29, *p* = .03. They had a higher mean item-based recall score (recalling more words from the large category) than the TD group (ASD: *M* = .62, *SD* = .16; TD: *M* = .53, *SD* = .10), although this difference marginally failed to reach significance at the .05 level, *F*(1, 26) = 3.77, *p* = .06. The interaction is illustrated in Figure 3.[Fig-anchor fig3]

#### EF measures

To assess performance in the ID/ED task, errors in the extradimensional shift block and total number of errors were analyzed. A univariate ANOVA on group (ASD vs. TD) revealed no group difference in either the extradimensional shift errors (ASD: *M* = 10.42, *SD* = 7.88; TD: *M* = 7.07, *SD* = 5.38), *F*(1, 26) = 1.73, *p* = .20, or total errors (ASD: *M* = 19.14, *SD* = 11.39; TD: *M* = 13.86, *SD* = 6.57), *F*(1, 26) = 2.26, *p* = .15.

Group performance on the SSP was assessed by analyzing span length (the longest sequence successfully recalled) using a univariate ANOVA. There was again no significant difference in performance between groups in this task (ASD: *M* = 5.50, *SD* = 1.34; TD: *M* = 6.14, *SD* = 0.95), *F*(1, 26) = 2.14, *p* = .16.

#### Correlational analysis

First, the relationships between the psychometric measures (BPVS and RPM) and performance on the memory and EF measures were analyzed. As in Experiment 1, Spearman’s correlation coefficients showed no significant relationships for any of the experimental tasks (*p*s > .05). We then assessed the role that EF plays in the recall of items from the small categories (thought to be reliant on relational memory processes) and the recall of items from the large category (thought to be reliant on item-based memory processes). In the ASD group, the proportion of items remembered from the small categories was significantly correlated with performance on the SSP task (*r* = .54, *p* = .05), but not correlated with performance on the ID/ED task (*r* = .41, *p* = .14). Recall of items from the large category was not correlated with either SSP (*r* = .05, *p* = .86) or ID/ED (*r* = .01, *p* = .97). The results indicated that good recall of the small categories, thought to reflect good relational memory, was associated with good visuospatial maintenance. There were no significant correlations between EF and performance on the category memory task in the TD group (*p*s > .64).

The children in the ASD group were then divided into high- and low-SSP-performance subgroups, depending on whether their SSP score fell below or above the median for the ASD group (*Mdn* = 5.5). The ASD children in the high-SSP subgroup (*n* = 7) were not significantly impaired in relational recall, retrieving a similar proportion of words from the small categories as the TD group (*n* = 14; ASD: *M* = .53, *SD* = .21; TD: *M* = .64, *SD* = .22), *t*(19) = 1.13, *p* = .272. In contrast, the ASD children in the low-SSP subgroup (*n* = 7) recalled a significantly smaller proportion of the small-category words (*M* = .35, *SD* = .25) compared with the TD children, *t*(19) = 2.70, *p* = .014. Neither ASD subgroup differed compared with TD group on age, RPM, or BPVS score (*p*s > .05).

## Discussion

Two experiments assessed the hypothesis that the relational memory impairment in autism results from a specific impairment in hippocampally mediated, automatic associative retrieval processes with an increased reliance on effortful, frontally mediated retrieval processes. In Experiment 1, children with ASD recalled fewer episodic autobiographical details and fewer items from a list of related words compared with control children. In contrast, children with ASD recalled more general autobiographical details and the same number of items from a list of unrelated words compared with control children. Using a tighter procedure that removed the possibility of supporting any particular memory strategy, Experiment 2 demonstrated analogous findings: Children with autism obtained lower relational memory scores by recalling fewer items from small categories of words than control children. This is consistent with the view that children with autism have a deficit in relational retrieval, because E.ABMs are embedded within a set of contextual information and thus require relational processing for their retrieval. In contrast, retrieval of general and factual information can be completed in a more item-based, nonrelational manner.

No group differences were found in any of the executive measures, suggesting that the relational memory impairment was not an effect of a general executive dysfunction in the ASD group. A correlational analysis revealed a strong association in the ASD group between two EF functions (specifically, visuospatial working memory and set-shifting ability) and relational memory measures. In contrast, the executive abilities of the ASD group were not associated with performance on the item-based memory measures. In the TD group, no relationships were found between executive ability and any of the memory measures. These results suggest that those individuals with ASD with good executive functioning are able to atypically employ certain executive processes to perform relational memory retrieval, which is normally reliant on rapid, automatic, associative processing.

A growing body of neuroscientific evidence has implicated the hippocampus (HC) as a critical structure in relational memory processes (e.g., Davachi, Mitchell, & Wagner, 2003; Davachi & Wagner, 2002; Eichenbaum, 2004; Eldridge, Knowlton, Furmanski, Bookheimer, & Engel, 2000), raising the possibility that the source of the relational memory deficit we observed here in ASD is hippocampal in origin for example, (Bowler et al., 2008b; Gaigg et al., 2008). However, Boucher and colleagues (Boucher & Mayes, 2012; Boucher et al., 2012) have argued against hippocampal abnormality on functional grounds: unlike individuals with hippocampal lesions, individuals with autism do not show marked deficits in cued recall or paired associate learning (PAL). Nonetheless, such findings are not unequivocal. For example, Brown and colleagues (Brown, Aczel, Jiménez, Kaufman, & Plaisted-Grant, 2010) found a subtle impairment in PAL even in high functioning children with ASD, and Gaigg, Rogers and Bowler (2012) have recently reported impaired PAL of complex stimuli in high functioning adults with ASD. Thus, the hippocampus remains an important candidate mechanism for the site of the relational memory impairment seen in autism, and requires further investigation.

A second possibility is that relational memory retrieval is compromised in ASD due to frontal lobe abnormality, which adversely impacts effortful memory processes such as specification of cues, thereby reducing the initiation of automatic associative activation of clusters of related items. This is consistent with our finding that those children with better EF functioning scores performed better on measures of relational learning, suggesting that ASD individuals with spared frontal and EF functions are able to strategically specify appropriate retrieval cues in order to initiate associative retrieval and thus show relatively good relational memory compared with those with compromised frontal and EF functioning. There is both neuroanatomical and behavioral evidence consistent with this view. For example, both individuals with ASD and frontal lobe patients show impaired free recall, but intact recognition (see Baldo & Shimamura, 2002, for review). However, more subtle and penetrating studies have suggested otherwise. For example, Bowler, Gaigg, and Gardiner (2010) found that individuals with ASD differed from a pattern of performance expected from studies of frontal patients, in showing typical levels of list learning, cued recall and memory interference. Furthermore, Kopelman and Stanhope (1998) showed that patients with frontal lesions showed disproportionate *benefit* in the recall of semantically categorized words, compared with unrelated words. Individuals with ASD are known to display the opposite pattern of recall; their recall performance shows little benefit from semantically organizable material, resulting in impairment relative to comparison participants. Thus, it seems unlikely that a frontal deficit alone can explain the selective relational memory deficit observed in the current study.

A third possibility is compromise in ASD to a network comprising the HC, prefrontal cortex (PFC), and posterior parietal cortex (PPC) that supports relational memory. Boucher and colleagues have recently urged consideration of PPC in understanding memory impairments in autism (Boucher & Mayes, 2012; Boucher et al., 2012). A newly emerging field of memory research argues for an important role of PPC in episodic and ABM (Berryhill, Phuong, Picasso, Cabeza, & Olson, 2007; Simons et al., 2008; Simons, Peers, Mazuz, Berryhill, & Olson, 2010; Wagner, Shannon, Kahn, & Buckner, 2005). Further, experimental studies have demonstrated that both patients with PPC lesions (Davidson et al., 2008) and individuals with ASD (Bowler, Gardiner, & Gaigg, 2007) give fewer “remember” responses during the remember/know task, thought to reflect reduced autonoetic consciousness during retrieval. Boucher and colleagues (Boucher & Mayes, 2012; Boucher et al., 2012) have recently argued that this region may be more likely to be the critical site of impairment than HC on the basis of their argument regarding reports of preserved cued recall and PAL in ASD, and further that PPC, along with PFC and HC, is involved in the default mode network (DMN; Buckner, Andrews-Hanna, & Schacter, 2008, although see Fornito, Harrison, Zalesky, & Simons, 2012), which is thought to be atypical in ASD as a consequence of reduced connectivity between the subsystems of the DMN (Anderson et al., 2011; Cherkassky, Kana, Keller, & Just, 2006). Thus, our finding of a selective correlation between measures of EF functions and relational, but not item-specific, memory in ASD can be accounted for by reduced connectivity between a relational memory network of HC, PFC, and PPC. Compromise to this network could require greater or prolonged activation of ventrolateral PFC involved in iterative specification of retrieval cues following failed relational memory search and/or prolonged evaluation of poorly specified retrieved information for verification (see Dobbins, Foley, Schacter, & Wagner, 2002, and Simons & Spiers, 2003, for mnemonic functions of ventrolateral PFC), resulting in the correlation between relational memory and executive measures only in the autistic group.

Alternatively, it may be less the integrity of the relational memory network or its subsystems that underlies our effect, but rather differences in the nature of the input representations to the network held within the neocortical memory system. At least two possibilities present themselves. One is the notion of enhanced local processing in ASD. Superior ASD performance has been observed in perceptual, attentional, and linguistic tasks requiring the processing of isolated items or features, reflecting a bias in ASD toward processing item-based local information rather than relational and contextual information (e.g., Happé, 1999; Happé & Frith, 2006; Mottron, Dawson, Soulières, Hubert, & Burack, 2006; Plaisted, Saksida, Alcántara, & Weisblatt, 2003). Thus, it is possible that the relatively poor performance in relational memory tasks could reflect a bias toward item-based memory, rather than a relational memory deficit per se. The other possibility relates to the automatic associative retrieval component of Moscovitch’s (1992; Moscovitch & Winocur, 2002) model and is predicted by Plaisted’s (2001) reduced generalization theory. This theory states that autistic cognition is characterized by a reduced sensitivity to information that is held in common between different stimuli, and enhanced sensitivity to features unique to a stimulus. One consequence of this information-processing style is that information related by similarity is represented further apart in psychological space, resulting in reduced spread of associative excitation across a network of related information (see also Beversdorf, Narayanan, Hillier, & Hughes, 2007, for a similar theoretical approach). Plaisted (2001) has claimed that this is the cause of reduced categorization abilities (Alderson-Day & McGonigle-Chalmers, 2011; Bott, Brock, Brockdorff, Boucher, & Lamberts, 2006; Gastgeb, Rump, Best, Minshew, & Strauss, 2009; Gastgeb, Strauss, & Minshew, 2006; Plaisted, 2000; Vladusich, Olu-Lafe, Kim, Tager-Flusberg, & Grossberg, 2010) and, complementarily, enhanced discrimination skills frequently observed in ASD (e.g., Bonnel et al., 2003; Heaton, Hermelin, & Pring, 1998; Lepistö et al., 2005; Mottron et al. 2006; O’Riordan & Passetti, 2006; Plaisted, O’Riordan, & Baron-Cohen, 1998; Plaisted et al., 2003).

Both enhanced local processing (Happé & Frith, 2006; Mottron et al., 2006) and reduced generalization (Plaisted, 2000, 2001) predict that relational information will be less available to the HC-PFC-PPC memory network, but for different reasons. In the case of enhanced local processing, if item-based processing dominates over relational processing, then frontal recruitment may be necessary to inhibit enhanced item-based processing in order to allow relational information to be made available to the HC-PFC-PPC network. In contrast, reduced generalization directly predicts reduced activation of relational information. Frontal executive recruitment therefore reflects ventrolateral PFC activation in the service of greater specification of retrieval cues in order to rerun failed memory searches and/or evaluation of poorly specified retrieved information for verification.

The central difference between these two accounts is the role that each ascribes to the atypical use of EF in ASD in relational memory tasks observed here. Reduced generalization theory regards EF use as a compensation mechanism for the impoverished input of relational information into the memory retrieval system. In contrast, the enhanced local processing theory regards EF use as reflecting the suppression of item-based information that interferes and competes with relational information for access to the memory retrieval system. Indeed, the finding that retrieval of semantic ABM details in Experiment 1 was enhanced in our children with ASD, along with the numerically greater number of items recalled from large categories in Experiment 2, hints at this possibility that EF functions are employed to suppress enhanced item-based processing that otherwise interferes with memory dealing with relational information. However, the lack of relationship with the Stroop inhibition measure permits speculation that an alternative reason for enhanced item-specific recall is that this may be the primary output of a memory system that is unable to fluently activate associatively related information as a consequence of poor processing of information held in common between items. Item-specific information, therefore, by default, dominates memory recall. Future experiments employing more sensitive methodology and targeted experimental designs are needed to tease these potential mechanisms apart.

One possibility raised by both reduced generalization and enhanced local processing theories is that the integration of relational information during encoding may be impaired in ASD. This could result in an impoverished, poorly integrated memory trace, which would require the recruitment of additional effortful, executive processes at retrieval in order to successfully recall the relational information. This bears striking similarities to accounts of memory processing in older adults, which is characterized by poor relational memory but intact item-based memory (see Shing et al., 2010, for review). A number of studies have shown that older adults recruit effortful, frontal processes when retrieving relational, but not item-based, information, and intriguingly, this effortful processing appears to be less necessary in young adults (e.g., Kuo & Van Petten, 2006; Swick, Senkfor, & Van Petten, 2006). For example, Kuo and Van Petten (2006) showed that older adults’ effortful recruitment of executive processing during retrieval was less evident when support had been provided at encoding, which directed processing resources to relational information. They concluded that executive, frontal mechanisms are engaged by the demand to retrieve weakly encoded relationships. It is therefore possible that children with ASD show reduced integration of relational information during encoding, whether it is due to reduced generalization or enhanced local processing, and thus need to recruit additional, effortful processes in order to recall the relational information during retrieval. In contrast, TD children may integrate relational information into memory more successfully, and thus are able to rely on more automatic, associative retrieval processes during recall.

This may have interesting implications for the memory of self-relevant ABMs. A wide literature has reported differences and impairments in self-awareness and self-concept in individuals with ASD (see Lind, 2010, for review). For neurotypical individuals, the self is an important framework for structuring and organizing episodic and ABMs (Conway & Pleydell-Pearce, 2000), and may provide a context for personal experience that gives rise to autonoesis, or a “feeling of reexperiencing” (Wheeler, Stuss, & Tulving, 1997). If individuals with ASD have diminished self-awareness, event information may only be poorly integrated into a self-framework during memory encoding (Lind, 2010; Lind & Bowler, 2010). This may result in poorer retrieval of E.ABMs, a more heavy reliance on general and semantic facts, and a lack of autonoesis, or feeling of reexperiencing, all of which have now been reported in ASD (e.g., Bowler et al., 2000; Crane & Goddard, 2008).

There are some limitations to the present study that are important to note. First, the significant correlations revealed between EF ability and relational memory in the ASD groups may not necessarily suggest that the children with ASD were employing executive strategies for relational memory processing; it is possible that these relationships are mediated by a third, untested factor. However, controlling for verbal ability, which was identified as a likely mediating factor, did not change the outcome of the correlational analyses. Second, the word lists used in the category memory task in Experiment 2 were not explicitly matched on average frequency of response, with the large category containing words with a lower average frequency than the small categories. However, this confound between size of category and frequency of response would predict better retrieval of the items from the small category list, which is exactly the opposite of what was found in the ASD group. Third, although there were no significant differences in performance between ASD and TD groups on any of our EF measures, the children with ASD sometimes performed at a numerically lower level than the TD children. However, these numerical differences were small, and, furthermore, a general executive dysfunction account does not appear to be able to explain the selective association between executive processes and relational memory tasks or the spared and often superior performance shown in item-based memory tasks. Finally, our study used relatively small samples of high-functioning children, and thus results should be interpreted with caution and may not extend to lower-functioning individuals. However, the general findings of EF recruitment during relational memory tasks in ASD children was replicated in two experiments using three different memory tasks, and thus we believe the findings may be robust in the high-functioning population.

Nonetheless, the findings of the current study have important implications for the way we understand memory in ASD. If children with ASD recruit EF functions to perform relational memory tasks, this suggests that processing relations in memory is more effortful for them than it is for TD children. Further, individual variability in executive abilities in ASD may affect how well tasks involving relational information are performed. Individuals with good EF functions may perform relational tasks at typical levels; however, individuals with poor EF functions may be impaired relative to TD individuals. If this employment of EF functions to process relational information is not restricted to memory, but is also involved in online tasks in which contextual relationships between items must be processed, this may have important consequences for how we understand cognition in ASD as a whole. If the ASD group employed EF functions during tasks that the TD group performed more automatically, this suggests that poor EF functions may affect ASD performance even on tasks not typically thought to be reliant on executive processing. This raises the possibility that executive *dys*function in ASD, when present, may have an even more pervasively detrimental effect on cognition than it may in other populations.

Overall, the results of the current study suggest that individuals with ASD employ effortful, executive processes in order to process relational information in memory. This has important implications for the implementation of interventions and teaching in educational settings. Given the demanding executive nature of relational memory tasks for children with ASD, simply minimizing other executive demands during encoding and retrieval of relational information may increase the executive resources available to maximize relational and contextual processing. Further, the explicit cueing of attention toward relational information in a learning setting may enable children with ASD to encode, retrieve, and learn about relational information in a typical way, as cueing may alleviate the executive burden required to suppress a more dominant item-based memory system. Finally, given the effort required to process relational information, it is important to consider the possibility that children with ASD may require higher levels of motivation to maximize performance on relational tasks than that may be needed by TD children.

## Figures and Tables

**Table 1 tbl1:** Mean Age and Psychometric Scores for the Typically Developing Group and the Autism Spectrum Disorder Group

Measure	TD group (*n* = 14)		ASD group (*n* = 14)		Group differences	
	*M*	*SD*	*M*	*SD*	*F*(1, 26)	*p*
Chronological age (years)	12.1	0.2	12.2	0.6	1.31	.26
Standardized BPVS	120.6	19.7	109.5	13.7	2.99	.10
RPM	46.4	7.4	43.9	6.4	0.91	.35
*Note*. TD = typically developing; ASD = autism spectrum disorder; BPVS = British Picture Vocabulary Scale; RPM = Raven’s Standard Progressive Matrices.						

**Table 2 tbl2:** Mean Age and Psychometric Scores for the Typically-Developing Group and the Autism Spectrum Disorder Group

Measure	TD group (*n* = 14)		ASD group (*n* = 14)		Group differences	
	*M*	*SD*	*M*	*SD*	*F*(1, 26)	*p*
Chronological age (years)	11.8	1.1	11.8	1.4	0.03	.87
Standardized BPVS	110.1	13.1	110.3	13.6	<0.01	.98
RPM	44.3	6.3	41.6	8.3	0.95	.33
*Note*. TD = typically developing; ASD = autism spectrum disorder; BPVS = British Picture Vocabulary Scale; RPM = Raven’s Standard Progressive Matrices.
